# D-Fructose Assimilation and Fermentation by Yeasts Belonging to Saccharomycetes: Rediscovery of Universal Phenotypes and Elucidation of Fructophilic Behaviors in *Ambrosiozyma platypodis* and *Cyberlindnera americana*

**DOI:** 10.3390/microorganisms9040758

**Published:** 2021-04-05

**Authors:** Rikiya Endoh, Maiko Horiyama, Moriya Ohkuma

**Affiliations:** Microbe Division/Japan Collection of Microorganisms, RIKEN BioResource Research Center (RIKEN BRC-JCM), 3-1-1 Koyadai, Tsukuba, Ibaraki 305-0074, Japan; maiko.horiyama@riken.jp (M.H.); mohkuma@riken.jp (M.O.)

**Keywords:** physiology, physiological characterization, ascomycetous yeasts, fructophily, Kluyver rule

## Abstract

The purpose of this study was to investigate the ability of ascomycetous yeasts to assimilate/ferment d-fructose. This ability of the vast majority of yeasts has long been neglected since the standardization of the methodology around 1950, wherein fructose was excluded from the standard set of physiological properties for characterizing yeast species, despite the ubiquitous presence of fructose in the natural environment. In this study, we examined 388 strains of yeast, mainly belonging to the Saccharomycetes (Saccharomycotina, Ascomycota), to determine whether they can assimilate/ferment d-fructose. Conventional methods, using liquid medium containing yeast nitrogen base +0.5% (*w/v*) of d-fructose solution for assimilation and yeast extract-peptone +2% (*w/v*) fructose solution with an inverted Durham tube for fermentation, were used. All strains examined (*n* = 388, 100%) assimilated d-fructose, whereas 302 (77.8%) of them fermented d-fructose. In addition, almost all strains capable of fermenting d-glucose could also ferment d-fructose. These results strongly suggest that the ability to assimilate/ferment d-fructose is a universal phenotype among yeasts in the Saccharomycetes. Furthermore, the fructophilic behavior of *Ambrosiozyma platypodis* JCM 1843 and *Cyberlindnera americana* JCM 3592 was characterized by sugar consumption profiles during fermentation.

## 1. Introduction

Physiological tests have long been utilized to characterize yeast species with poor morphological traits. As is similar to the situations in taxonomic studies for the majority of bacteria, physiological properties have been a major feature in distinguishing and identifying yeast species until the era of molecular phylogeny. Although the importance of molecular phylogeny is widely accepted in the field of the systematics of microbes, physiological characterization has been an important aspect of new taxon descriptions. A set of common sugars, alcohols, sugar alcohols, and organic acids has been routinely used for assimilation tests of carbon compounds for yeasts. The fundamental methodology of carbon assimilation test for yeasts was published more than 70 years ago [1] and then standardized in the monograph “*The Yeasts, a Taxonomic Study*” [2]. In the latest version of the monograph, “*The Yeasts, a Taxonomic Study, 5th edition”*, published in 2011 [3], assimilation of 36 carbon compounds was routinely profiled for almost all ascomycetous yeast species. However, **d**-fructose was still not included.

Fructose is a ketonic C6-monosaccharide naturally found in many plants. For instance, grapes are a rich source of sugars, containing equal amounts of glucose and fructose, and their total hexose content typically ranges from 160 to 300 g·L^−1^ [4]. The ability of *Saccharomyces cerevisiae* to ferment fructose was well documented in the early 20th century [5]. The presence of fructose in the natural environment, such as in fruits, and its importance as a carbon source have been highlighted in some previous studies, particularly in the field of food microbiology. For instance, fructose serves as the carbon source metabolized by yeasts during grape spoilage [6,7] or wine fermentation [8]. The fructophilic yeasts *Zygosaccharomyces rouxii* and *Z. bailii* are involved in canned fruit or fruit juice spoilage [9]. The characteristic fructophilic behavior of *Zygosaccharomyces* species is associated with the presence of the fructose facilitator *Zygosaccharomyces* genes, which encode hexose transporters [10]. Despite its ubiquitous presence in the natural environment and despite knowledge of its potential utility as a substrate for fermentation, fructose has not been included in the standard set of assimilation of carbon compounds since the first publication of the monograph, “*The Yeasts, a Taxonomic Study”*. Consequently, there is still limited data available on the ability of yeasts to assimilate/ferment fructose.

According to Wickerham and Burton (1948) [1] and Miller and Phaff (1958) [11], carbon assimilation tests were perhaps first applied to yeasts by Beijerinck in 1889 [12]. The methods were reexamined by Wickerham and Burton 1948 [1], and then well-establish in the monograph “*The Yeasts, a Taxonomic Study*” [2], which has long been accepted as the gold standard of characterization for yeasts. Wickerham and Burton 1948 [1] mentioned—“Up to the present time the carbon sources used in assimilation tests in the major attempts at yeast classification have been limited to glucose, fructose, mannose, …” However, fructose was not included in the standard set of carbon assimilation tests in “*The Yeasts, a Taxonomic Study*” (1952) [2]. This monograph mentioned that tests with fructose had been omitted because, during many years, experiences have taught us that the rule, first formulated by Kluyver, according to which a yeast able to ferment glucose can also ferment fructose and mannose, holds good without a single exception [2] (p. 22); perhaps the “Kluyver” mentioned herein would designate the literature of Kluyver (1912) [13]. Due to this exclusion of fructose from the standard set, studies on fructose fermentation/assimilation have been intermittent since then. In 1985, Konno et al. examined the so-called Kluyver rule using over 200 yeast type strains and reported the results very briefly on half of one page [14]. The authors mentioned that the “Kluyver rule” was generally true, with a special emphasis on *Torulopsis halophila* (current name: *Wickerhamiella versatilis*; the strain used in the study was not specified) that was negative for fructose fermentation, despite being positive for glucose fermentation. It is very disappointing that the materials and methods were not described in detail [14]. Therefore, very little detailed information is available about the previous examination of the “Kluyver rule”.

In the course of phenotypic quality control of yeast strains in our culture collection at Microbe Division/Japan Collection of Microorganisms, RIKEN BioResource Research Center (RIKEN BRC-JCM), we confirmed that many strains actually had the ability to ferment fructose. This led to the survey of the capability of a wide variety of yeasts in the Saccharomycetes (Saccharomycotina, Ascomycota) to assimilate/ferment fructose, namely reexamination of the “Kluyver rule” by using JCM strains. Thus, the purpose of this study was to identify the range of yeast species that are capable of utilizing fructose. We also reexamined their ability to assimilate/ferment sucrose. Sucrose is a common disaccharide that can be hydrolyzed by invertase to glucose and fructose [15]. The ability of glucose and sucrose assimilation or fermentation has been examined in almost all known ascomycetous yeast species. Specifically, in the present study, the generality of fructose assimilation or fermentation was evaluated by comparing their positive percentages.

As will be presented hereafter in this paper, universal phenotypes of “fructose assimilation” and “fructose fermentation” of yeasts in the Saccharomycetes (Saccharomycotina, Ascomycota) were rediscovered in this study. The reasons underlying the lack of information about the phenotypes have also been discussed. In addition, fructose and glucose consumption in the fermentation liquid media by two yeast strains, *Ambrosiozyma platypodis* JCM 1843 and *Cyberlindnera americana* JCM 3592, was monitored as they exhibited specific fructophilic behaviors. Please note that the term “fructose” in the present paper always indicates **d**-fructose.

## 2. Materials and Methods

### 2.1. Yeast Strains

Yeast strains used in this study were obtained from RIKEN BRC-JCM. The strains examined for the assimilation/fermentation tests are listed in Table 1. Strain information, including the voucher numbers, isolation source, and GenBank accession numbers of the reference nucleotide sequence, is described in the online strain catalog of RIKEN BRC-JCM (https://jcm.brc.riken.jp/en/catalogue_e; 16 March 2021). Most of them belonged to the Saccharomycetes (Saccharomycotina, Ascomycota). A few species in the genus *Schizosaccharomyces* and *Saitoella complicata* in Taphrinomycotina and *Trichosporiella flavificans* in Pezizomycotina were also employed. The yeast strains were incubated at 25 °C for precultivation, assimilation, and fermentation, with the exception of *Cyberlindnera rhizosphaerae* JCM 16499 (8 °C), *Debaryomyces coudertii* JCM 2387 (15 °C), *Kazachstania telluris* JCM 5298 (37 °C), and *Wickerhamomyces patagonicus* JCM 16381 (15 °C).

### 2.2. Assimilation of Fructose

The assimilation of fructose was examined using the conventional method for yeast identification [3]. Experiments on fructose and sucrose assimilation were performed twice independently using commercially available highly pure reagents obtained from the two different suppliers. Briefly, an aqueous stock solution containing 6.7% (*w/v*) yeast nitrogen base (YNB, Difco Labs, Thermo Fisher Scientific, Waltham, MA, USA, 239210) and 5% (*w/v*) fructose (Nacalai Tesque, Inc., Kyoto, Japan, GR grade, cat. 16315-55; FUJIFILM Wako Chemical Corporation, Miyazaki, Japan, GR grade, cat. 147-02765) was filter-sterilized, and 0.2 mL of the sterilized stock solution was mixed with 1.8 mL of sterile distilled water in a sterile glass test tube to prepare a working liquid medium containing 0.67% (*w/v*) YNB and 0.5% (*w/v*) fructose. Glucose (Nacalai Tesque, Inc., GR grade, cat. 16806-25) or sucrose (Nacalai Tesque, Inc., GR grade, cat. 30404-45; Kanto Chemical Co.,INC., Tokyo, Japan, GR grade, cat. 37000-01) were also employed instead of fructose in the above-mentioned assimilation medium as a reference. A plain YNB solution was used as a negative control. Yeast culture was prepared on YM agar (2.1% (*w/v*) of YM broth (Difco Labs., Thermo Fisher Scientific, 271120) plus 2% (*w/v*) agar (Nacalai Tesque, Inc., cat. 01028-85)) 2–7 days before inoculation and a vigorously grown culture was inoculated into the liquid media. Growth was visually monitored and scored weekly for up to four weeks. Growth was measured according to the above-mentioned monograph with some modifications [3]. Briefly, the degree of growth in the liquid medium was observed by the naked eye after shaking the test tube to disperse the yeast cells. The test tube was placed on a white file card, on which 0.75 mm thick black lines were drawn at intervals of approximately 5 mm. The results were scored as 3+ when the lines were completely obscured, 2+ when the lines appeared as diffused bands, 1+ when the lines were distinguishable but had blurred edges, and negative when the lines were distinct with sharp edges. The results were as follows: Strongly positive (3+ reading developed within 1 week), positive (2+ or 3+ reading developed within 2 weeks), slowly positive (2+ or 3+ reading developed slowly over a period exceeding two weeks), delayed positive (2+ or 3+ reading developed rapidly but after two weeks), weakly positive (1+ reading developed), and negative (little (less than 1+ reading) or no growth).

### 2.3. Fermentation of Fructose

Fermentation of fructose was also examined by the conventional method for yeast identification [3]. Experiments on fructose and sucrose fermentation were performed twice independently using commercially available highly pure reagents obtained from the two different suppliers. Briefly, 4.5 mL of sterile fermentation basal medium containing 0.45% (*w/v*) bacto yeast extract (Difco Labs., Thermo Fisher Scientific, 212750), 0.75% (*w/v*) bacto peptone (Difco Labs., 211677), and ~50 ppm bromothymol blue (Sigma-Aldrich, St. Louis, MO, USA, B8630) was prepared in a glass test tube with a small, inverted Durham tube inside. An aqueous stock solution of 20% (*w/v*) fructose (Nacalai Tesque, Inc., GR grade, cat. 16315-55; FUJIFILM Wako Chemical Corporation, GR grade, cat. 147-02765) was filter-sterilized, and 0.5 mL of the sterilized stock solution was added to the fermentation basal liquid medium to obtain a final concentration of 2% (*w/v*) fructose. Glucose (Nacalai Tesque, Inc., GR grade, cat. 16806-25) or sucrose (Nacalai Tesque, Inc., GR grade, cat. 30404-45; Kanto Chemical Co.,INC., GR grade, cat. 37000-01) were also employed instead of fructose in the above-mentioned fermentation medium as a reference. Yeast culture was prepared in the same manner as for assimilation tests, and a vigorously grown culture was heavily inoculated into the liquid media. Filling with gas in the inverted tube (Supplementary Figure S1) was visually monitored and scored about every second day up to one week and then at two and three weeks after inoculation. The results were scored according to the above-mentioned monograph with some modifications as follows [3]: Strongly positive (the Durham tube rapidly filled with gas within three days), positive (more than half of the Durham tube filled with gas within seven days), slowly positive (more than half of the Durham tube filled with gas after more than seven days), delayed positive (more than half of the Durham tube rapidly filled with gas, but only after more than seven days), weakly positive (less than half of the Durham tube filled with gas), or negative (no gas accumulation observed in the Durham tube).

### 2.4. Sugar Consumption during Fermentation

Sugar consumption by *A. platypodis* JCM 1843, *C. americana* JCM 3592, and *S. cerevisiae* JCM 7255 in the fermentation liquid media was monitored, as the former two strains exhibited apparent fructophilic behaviors during fermentation (see Section 3.2.).

Fermentation liquid media were prepared in the same manner as described in Section 2.3 with the following modifications. The total amount of medium was 7 mL in a glass test tube to allow a series of liquid medium sampling, and bromothymol blue was not added to the media to avoid interference with absorbance at 340 nm in the subsequent measurement using a spectrophotometer. Three kinds of fermentation media were prepared: (i) 2% (*w/v*) fructose, (ii) 2% (*w/v*) glucose, and (iii) 2% (*w/v*) fructose plus 2% (*w/v*) glucose (final concentrations in the media). To prepare the fructose–glucose mixed medium (iii), an aqueous stock solution of 20% (*w/v*) fructose (Nacalai Tesque, Inc.) plus 20% (*w/v*) glucose (Nacalai Tesque, Inc.) was filter-sterilized, and then 0.7 mL of the sterilized stock solution was added to 6.3 mL of the fermentation basal liquid medium.

Strains JCM 1843, JCM 3592, and JCM 7255 were cultured on YM agar at 25 °C for 2–3 days, and the freshly prepared culture was incubated in the basal fermentation medium at 25 °C for 2 days. The three fermentation media (i), (ii), and (iii) were inoculated with the culture and incubated at 25 °C without shaking. Inoculation was done in quadruplicates. The fermentation medium was sampled after gentle mixing by pipetting at approximately 12 h intervals for JCM 7255 and approximately 12–48 h intervals for JCM 1843 and JCM 3592. The sampled media were centrifuged to remove cells, and the supernatant was heated at 90 °C for 10 min to deactivate enzymes and then stored at −20 °C for measuring the fructose and glucose concentrations.

The concentration of fructose and glucose in the fermentation media was measured and calculated using an enzymatic test kit **d**-glucose/**d**-fructose (Boehringer Mannheim/R-Biopharm, Darmstadt, Germany, cat. 10 139 106 035) following the manufacturer’s instructions with some modifications. The absorbance of the solution in a 96-well microplate was measured at 340 nm using a spectrophotometer Multiskan SkyHigh (Thermo Fisher Scientific).

## 3. Results

### 3.1. Assimilation of Fructose

All 388 strains tested had the ability to assimilate fructose as well as glucose, utilizing fructose as the sole carbon source (Table 1). Sucrose was assimilated by fewer yeast strains than those capable of assimilating fructose; 229 (59.0%) out of the 388 strains assimilated sucrose (including strains of positive reaction delayed, slowly, and weakly positive).

### 3.2. Fermentation of Fructose

Three hundred and two (77.8%) out of the 388 strains had the ability to ferment glucose, and most of these strains could also ferment fructose (Table 1), with the exception of *Sporopachydermia quercuum* JCM 9486, which fermented glucose but not fructose. In contrast, *Ambrosiozyma platypodis* JCM 1843 showed a stronger and quicker positive reaction to fructose than to glucose. A preference for fructose was also observed; *Cyberlindnera americana* JCM 3592 fermented fructose well, but not glucose. Thus, 302 (77.8%) of the 388 strains had the ability to ferment fructose. These observations of JCM 9486, JCM 1843, and JCM 3592 were reproduced in three independent trials (Supplementary Table S1).

Similar to the results of assimilation, the number of strains capable of fermenting sucrose (99 strains) was much lower than that of strains capable of fermenting glucose/fructose. In addition, all the strains fermenting sucrose were capable of fermenting both glucose and fructose.

### 3.3. Sugar Consumption during Fermentation

**Figure 1:**
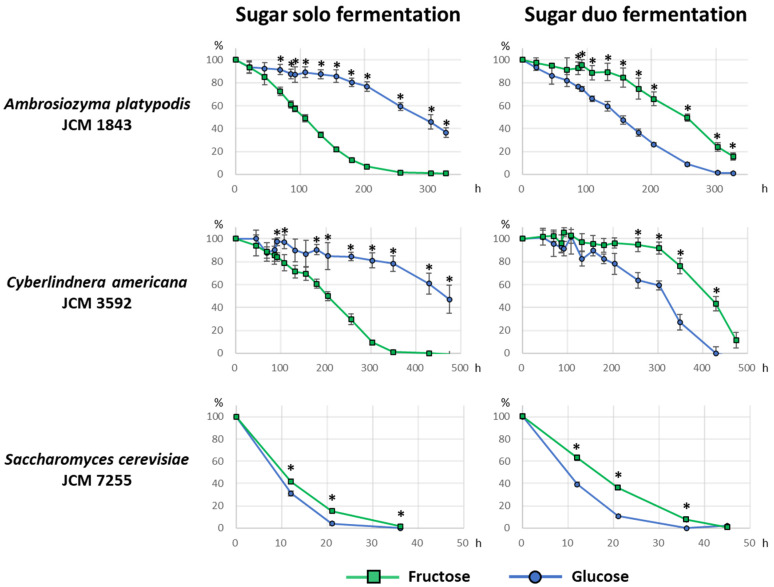
Sugar consumption profiles in the fermentation liquid media. The green square and blue circle indicate the percentage of fructose and glucose concentrations, respectively, compared with the initial amount of each sugar at 0 h. The left three graphs show sugar consumption by yeasts incubated in only 2% fructose or 2% glucose in the fermentation media. The right three graphs show sugar consumption by yeasts incubated in the fermentation medium containing both 2% fructose and 2% glucose. The bar on the symbols indicates standard deviation. Asterisk (*) indicates a significant difference between fructose and glucose percentages (Welch’s *t*-test, *p* < 0.01).

Sugar consumption by *A. platypodis* JCM 1843 and *C. americana* JCM 3592 in the fermentation liquid media was monitored using only 2% fructose or 2% glucose (sugar solo fermentation), or both 2% fructose and 2% glucose (sugar duo fermentation). Figure 1 shows the time-course consumption profiles of fructose and glucose, where the amount of sugars at 0-h (sampled immediately after inoculation) was set as 100%.

In *A. platypodis* JCM 1843 and *C. americana* JCM 3592, the sugar consumption profiles were similar to each other in both sugar solo fermentation and sugar duo fermentation. Fructose was more rapidly consumed than glucose in sugar solo fermentation. On the contrary, fructose consumption was substantially slower in sugar duo fermentation than in sugar solo fermentation; instead, glucose consumption was observed before fructose consumption.

*Saccharomyces cerevisiae* JCM 7255 rapidly consumed both fructose and glucose, which were almost used up at 36 h in both sugar solo/duo fermentation. Glucose consumption was always more rapid than fructose consumption. Fructose consumption in sugar duo fermentation was apparently less rapid than in sugar solo fermentation.

## 4. Discussion

The results of this study are very simple; all the yeast strains tested could assimilate glucose, and glucose fermenters were fructose fermenters, with a few exceptions. This strongly suggests that the utility of fructose is universal among yeasts in the Saccharomycetes. Positive reactions in assimilation and fermentation of fructose should be regarded as universal phenotypes rediscovered by this survey. We employed approximately 380 species of yeasts belonging to the Saccharomycetes. This accounts for almost one-third of the described ascomycetous yeast species. As we used a taxonomically wide variety of yeasts, there is no doubt about the generality of the positive reactions in the fructose assimilation/fermentation tests, at least for the Saccharomycetes. Thus, the “Kluyver rule” was confirmed on the whole.

We searched the capability of assimilation/fermentation of other common sugars by ascomycetous yeasts, based on the data in the monograph “*The Yeasts, a Taxonomic Study, 5th edition*” [3] (Table 2). As shown in Table 2, the percentage of assimilating/fermenting sucrose was 59.0%/25.5% in this study, whereas it was 60.7%/24.2% in the monograph, suggesting that the selection of yeast species employed in this study was unbiased. Judging from the higher positive percentages for both assimilation and fermentation of fructose compared to those of the other sugars, it is safe to say that fructose is an easy-to-use carbon source for the yeasts.

The ability to ferment fructose by brewer’s yeast was well-known as early as in the first half of the 20th century, particularly in the context of “selective fermentation” observed in a mixture of glucose and fructose ([5,16] and the literature cited therein). However, yeast taxonomists have not paid serious attention to fructose. To the best of our knowledge, no recent work, except one, has employed fructose to characterize new yeast species [17]. In a recent publication, fructose was used to prepare an enrichment medium for the isolation of highly osmotolerant yeasts from natural substrates, as its solubility is much higher than that of glucose; unfortunately, assimilation/fermentation of fructose was not determined in the characterization of new species [18].

Why have such simple phenotypes largely neglected until now? The reasons would be: (1) fructose was not selected in the standard set of physiological characterization throughout the monograph “*The Yeasts, a Taxonomic Study*”; (2) physiological profile has been used mostly just as a key for yeast taxonomy; thus, fructose has been out of focus even though it occurs abundantly in the natural environment, such as in honey and fruits. Probably due to such a historical background, most of the recent researchers excluded physiological tests of fructose from the description of new yeast species, likely without paying attention to the “Kluyver rule”.

In the present work, some strains exhibited fructophily during fermentation, preferring fructose, as a substrate for fermentation, to glucose. As stated in the results section, *A. platypodis* JCM 1843 and *C. americana* JCM 3592 appeared to be fructophilic in the regular fermentation test (Table 1). Furthermore, JCM 1843 and JCM 3592 demonstrated a fructophilic behavior, as determined by sugar consumption profiles in sugar solo fermentation, and this is contradictory to the pattern of sugar consumption by *S. cerevisiae* JCM 7255, which always preferred glucose to fructose (Figure 1). Initially, we hypothesized that JCM 1843 and JCM 3592 might exhibit a fructophilic behavior even in sugar duo fermentation (mixed fermentation), similar to *Zygosaccharomyces* species [19]. However, to our surprise, fructose consumption appeared to be suppressed in sugar duo fermentation (Figure 1). It is remarkable that glucose fermentation by JCM 1843 and JCM 3592 seemed to be activated by the presence of fructose in the medium. To the best of our knowledge, this is a new “irregular” pattern of sugar consumption profile. Further molecular biological investigations are required to clarify the mechanism underlying this phenomenon.

In contrast to *A. platypodis* and *C. americana*, *S. quercuum* JCM 9486 exclusively prefers glucose over fructose, and this is similar to the case reported previously for *W. versatilis* [14]. The reason for this exception, however, remains unknown. These specific preferences of sugar may be related to their lifestyles in the natural environment, which is a fascinating research theme from the viewpoint of yeast ecology. For instance, *A. platypodis* and *C. americana* likely inhabit a fructose-rich environment, and therefore, possess potent fructose transporter(s). Furthermore, the fructophilic behavior in *A. platypodis* and *C. americana* would be adaptive to such an environment.

*Zygosaccharomyces rouxii* and *Z. bailii* were reported to be fructophilic [19], although a clear fructophilic reaction was not observed in our simple experiments. In a previous study, *Z. bailii* was found to first ferment fructose and then glucose in a medium containing both glucose and fructose [19]. The experimental conditions in the present study differed from those in the previous one. As the fermentation test was performed using either glucose or fructose separately in the present study, the priority of sugar utilization remained unknown. Additional yeast strains exhibiting a fructophilic phenotype may be found if fermentation tests using a medium containing both glucose and fructose are performed. Later, fructose transporters in the plasma membrane of the *Zygosaccharomyces* yeasts were studied with molecular biological interests in their fructophilic behavior [20,21,22]. In addition, the mechanism of fructose fermentation has been well investigated on a molecular basis in the wine yeast *S. cerevisiae* [4]. *S. cerevisiae* contains at least 20 transporters associated with hexose uptake [23]. Glucose uptake is facilitated by hexose transporters [24]. Following its uptake into the cell cytoplasm, glucose is phosphorylated to glucose-6-phosphate, subsequently isomerized to fructose-6-phosphate, and finally metabolized through the glycolytic pathway [25]. Fructose is transported by the hexose transporter (HXT) family of proteins [26] and directly phosphorylated to fructose-6-phosphate by hexokinases, such as Hxk1 and Hxk2 [27]. Our data indicate that most of the yeasts belonging to the Saccharomycetes would exhibit fructose transporters and express specific hexokinases that metabolize fructose to fructose-6-phosphate. Indeed, a novel proton-coupled fructose transporter, Frt1, has been identified in *Kluyveromyces lactis* [28]. The mechanism of fructose uptake and its subsequent metabolism would be further investigated from the viewpoint of molecular biology using a wider variety of ascomycetous yeast species. Novel fructose transporters may be identified by exploring *FRT1* gene analogs using the draft genome sequences of ascomycetous yeasts.

In this study, we aimed to survey a wide variety of yeast species belonging to Saccharomycetes; thus, a single strain of each species was tested for its ability to assimilate and ferment fructose, except *A. platypodis* (JCM 1843 and JCM 1796) and *C. americana* (JCM 3592 and JCM 3593). Although the fructophilic behavior was less apparent in JCM 1796 and JCM 3593 than in JCM 1843 and JCM 3592, respectively (Table 1), both *A. platypodis* and *C. americana* preferably fermented fructose. Additional reference strains should be surveyed for fructose assimilation and fermentation, particularly for *A. platypodis*, *C. americana*, *S. quercuum*, and *W. versatilis,* to conclude the exceptions are species-specific. In addition, we should examine the assimilation/fermentation profiles of basidiomycetous yeasts in future studies to determine whether fructose assimilation/fermentation is a universal phenotype in yeasts irrespective of their taxonomic position.

Lastly, it is suggested that tests for fructose should be resurrected in the standard set of physiological characterization for yeasts in the Saccharomycotina subphylum in order not to miss the special characteristics of yeasts.

## Figures and Tables

**Table 1:** Fructose assimilation and fermentation profiles of Saccharomycetes yeasts.

Species	JCM no.	Assimilation			Fermentation		
		Glucose	Fructose	Sucrose	Glucose	Fructose	Sucrose
**Saccharomycetes, Saccharomycotina**							
**Saccharomycetales**							
**Cephaloascaceae**							
*Cephaloascus fragrans*	7613	+	+	-	-	- ^1^	- ^1^
**Debaryomycetaceae**							
*Candida aaseri*	1689	+	+	+/s	-	-	-
*Candida albicans*	1542	+ST	+ST	+ST	+ST	+ST	-
*Candida atlantica*	9548	+ST	+ST	+ST	w/-	w/-	-
*Candida atmosphaerica*	9549	+ST	+ST	+ST	+	+	-
*Candida boleticola*	1500	+	+	-	+ST	+ST	-
*Candida buinensis*	9453	+ST	+ST	+ST	+ST	+ST	-
*Candida conglobata*	2373	+	+	-	+ST	+ST	-
*Candida dendronema*	1803	+	+	+	+ST	+ST	-
*Candida diddensiae*	9598	+	+	+	+	+	-
*Candida fluviatilis*	9552	+	+	+	+	+	-
*Candida friedrichii*	9553	+	+	+	+	+	-
*Candida glaebosa*	1590	+ST	+ST	+	-	- ^1^	- ^1^
*Candida insectamans*	9611	+	+	-	-	-	-
*Candida insectorum*	9457	+	+	+	+	+	-
*Candida lyxosophila*	7532	+	+	+	+ST	+	-
*Candida maltosa*	1504	+ST	+ST	+	+ST	+ST	+ST
*Candida multigemmis*	9559	+	+	+	+	+	-
*Candida oleophila*	1620	+	+	+	+ST	+ST	-
*Candida palmioleophila*	5218	+	+	+	-	- ^1^	- ^1^
*Candida membranifaciens*	9450	+ST	+ST	+ST	+	+	+
*Candida naeodendra*	1509	+	+	+	+ST	+ST	-
*Candida neustonensis*	14892	+ST	+ST	+ST	+	+	+/s
*Candida parapsilosis*	1612	+ST	+	+	+ST	+ST	-
*Candida pseudoglaebosa*	2168	+	+	+	+	+	-
*Candida saitoana*	1438	+ST	+	+	-	-	-
*Candida santamariae*	1816	+	+	-	+ST	+ST	-
*Candida schatavii*	1778	+ST	+ST	-	s	s	-
*Candida sojae*	1644	+	+	+	+ST	+ST	+
*Candida tammaniensis*	10730	+	+	+	+ST	+ST	-
*Candida thasaenensis*	17817	+	+	+	+ST	+ST	+
*Candida tropicalis*	1541	+ST	+ST	+ST	+ST	+ST	+ST
*Candida trypodendroni*	10731	+	+	+	+	+	-
*Candida psychrophila*	2388	+	+	-	-	- ^1^	- ^1^
*Candida viswanathii*	9567	+	+	+	+ST	+ST	-
*Candida xestobii*	9569	+	+	+	-	-	-
*Candida zeylanoides*	1627	+	+	-	-	-	-
*Danielozyma ontarioensis*	10729	+	+	+	+	+	+
*Debaryomyces coudertii*	2387	+	+	-	+	+	-
*Debaryomyces hansenii*	1990	+ST	+ST	+ST	+/s	+/s	+/s
*Debaryomyces maramus*	1528	+	+	+	w/-	w/-	w/-
*Debaryomyces nepalensis*	2095	+ST	+ST	+ST	+	+	+
*Debaryomyces prosopidis*	9913	+	+	+	+/s	s/w	s/w
*Debaryomyces udenii*	7855	+ST	+ST	+ST	+/d	+	d/w
*Kurtzmaniella fragi*	1791	+ST	+ST	+ST	+ST	+ST	-
*Kurtzmaniella natalensis*	1445	+	+	+	+ST	+ST	-
*Kurtzmaniella quercitrusa*	9832	+	+	+	+ST	+ST	-
*Lodderomyces elongisporus*	1781	+ST	+ST	+ST	+	+	-
*Meyerozyma guilliermondii*	10735	+ST	+ST	+ST	+	+	+
*Millerozyma acaciae*	10732	+ST	+ST	-	+ST	+ST	-
*Millerozyma farinosa*	10734	+ST	+ST	-	+ST	+ST	-
*Millerozyma koratensis*	12576	+ST	+ST	+ST	+	+	+
*Priceomyces carsonii*	8121	+ST	+ST	+	-	- ^1^	- ^1^
*Priceomyces castillae*	10733	+	+ST	-	-	-	-
*Priceomyces fermenticarens*	9589	+ST	+ST	-	-	-	-
*Priceomyces haplophilus*	1635	+ST	+ST	-	-	- ^1^	- ^1^
*Priceomyces medius*	10737	+	+	-	-	-	-
*Priceomyces melissophilus*	1707	+ST	+ST	+ST	-	- ^1^	- ^1^
*Scheffersomyces coipomensis*	8916	+ST	+ST	+	+ST	+ST	-
*Scheffersomyces ergatensis*	9599	+ST	+ST	+	+ST	+	-
*Scheffersomyces insectosa*	9842	+	+	+	+ST	+ST	-
*Scheffersomyces lignosum*	9837	+ST	+	+	+ST	+ST	-
*Scheffersomyces segobiensis*	10740	+	+	+	+ST	+ST	-
*Scheffersomyces shehatae*	9840	+	+	s	+ST	+ST	-
*Scheffersomyces spartiniae*	10741	+	+	+	+ST	+ST	-
*Scheffersomyces stipitis*	10742	+	+	+	+ST	+ST	-
*Schwanniomyces capriottii*	6177	+ST	+ST	+ST	+ST	+ST	+ST
*Schwanniomyces etchellsii*	3656	+ST	+ST	+ST	+ST	+ST	-
*Schwanniomyces occidentalis* var. *occidentalis*	8123	+ST	+ST	+ST	+ST	+	+
*Schwanniomyces occidentalis* var. *persoonii*	8127	+	+	+	+	+	+
*Schwanniomyces polymorphus* var. *africanus*	7443	+ST	+ST	+ST	+ST	+ST	+ST
*Schwanniomyces polymorphus* var. *polymorphus*	3647	+ST	+ST	+ST	+ST	+ST	+
*Schwanniomyces pseudopolymorphus*	3652	+ST	+ST	+ST	+ST	+ST	+ST
*Schwanniomyces vanrijiae*	3657	+ST	+ST	+ST	w	w	w
*Schwanniomyces yamadae*	6191	+	+	+	+ST	+ST	-
*Wickerhamia fluorescens*	1821	+	+	+	+ST	+ST	+
*Yamadazyma akitaensis*	10738	+ST	+ST	+ST	+	+	-
*Yamadazyma kitorensis*	31005	+ST	+ST	s	+	+	-
*Yamadazyma mexicana*	1835	+ST	+ST	+ST	+	+	-
*Yamadazyma nakazawae*	7529	+ST	+ST	+ST	+ST	+ST	-
*Yamadazyma philogaea*	10739	+	+	+	+	+	-
*Yamadazyma scolyti*	3654	+	+	+	+	+	-
*Yamadazyma takamatsuzukensis*	15410	+	+	+	+	+	-
*Yamadazyma tenuis*	9827	+ST	+	+	+	+	-
*Yamadazyma triangularis*	9449	+	+	+	w/-	w/-	-
*Yamadazyma tumulicola*	15403	+ST	+ST	+	+	+	-
**Dipodascaceae**							
*Dipodascus aggregatus*	31687	+	+	-	-	-	-
*Dipodascus australiensis*	31688	+	+	-	-	-	-
*Dipodascus eriense*	3912	+	+	-	+/s	w/-	-
*Dipodascus fermentans*	2468	+	+	-	+ST	+ST	-
*Dipodascus ingens*	9471	+	+	-	-	-	-
*Dipodascus ovetensis*	3706	+	+	-	-	-	-
*Dipodascus reessii*	1943	+	+	-	-	- ^1^	- ^1^
*Dipodascus tetrasperma*	6361	+ST	+ST	-	+ST	+ST	-
*Geotrichum rectangulatum*	1750	+ST	+	-	+ST	+ST	-
**Lipomycetaceae**							
*Babjevia anomala*	5988	+	+	-	-	- ^1^	- ^1^
*Lipomyces kononenkoae*	5989	+	+	+	-	-	-
*Lipomyces lipofer*	3769	+	+	+	-	-	-
*Lipomyces smithiae*	8928	+	+	+	-	- ^1^	- ^1^
*Lipomyces spencermartinsiae*	5990	+	+	+	-	-	-
*Lipomyces starkeyi*	5995	+	+	+	-	-	-
*Lipomyces suomiensis*	7660	+	+	-	-	- ^1^	- ^1^
*Lipomyces tetrasporus*	6000	+	+	+	-	- ^1^	- ^1^
*Myxozyma geophila*	5220	+	+	-	-	- ^1^	- ^1^
*Myxozyma kluyveri*	7661	+	+	w	-	- ^1^	- ^1^
*Myxozyma lipomycoides*	5198	+	+	-	-	- ^1^	- ^1^
*Myxozyma melibiosi*	5194	+	+	-	-	- ^1^	- ^1^
*Myxozyma mucilagina*	1834	+	+	+	-	- ^1^	- ^1^
*Myxozyma neglecta*	5197	+	+	-	-	- ^1^	- ^1^
*Myxozyma udenii*	8927	+	+	+	-	- ^1^	- ^1^
**Metschnikowiaceae**							
*Aciculoconidium aculeatum*	13354	+	+	+	s	s	-
*Clavispora fructus*	1513	+	+	-	+ST	+ST	-
*Clavispora lusitaniae*	7533	+	+	+	+ST	+ST	-
*Candida akabanensis*	9115	+ST	+ST	+ST	+ST	+ST	+ST
*Candida auris*	15448	+ST	+ST	+ST	+	+	+
*Candida haemulonii*	3762	+ST	+ST	+ST	+ST	+ST	+
*Candida intermedia*	1607	+ST	+	+	+ST	+ST	+ST
*Candida melibiosica*	9558	+	+	+	+ST	+ST	-
*Candida mogii*	1611	+	+	+	+ST	+ST	+ST
*Candida pseudointermedia*	1592	+ST	+ST	+ST	+ST	+ST	+ST
*Candida fukazawae*	1641	+	+	+	+ST	+ST	-
*Candida fungicola*	10142	+ST	+ST	+ST	-	-	-
*Candida mesenterica*	2368	+	+	+	-	-	-
*Candida musae*	1598	+	+	+	+ST	+ST	-
*Candida oregonensis*	1811	+ST	+ST	+	+ST	+ST	-
*Candida pseudohaemulonii*	12453	+ST	+ST	+ST	+ST	+	+
*Candida tsuchiyae*	1638	+ST	+ST	+ST	+ST	+ST	+
*Hyphopichia burtonii*	3708	+	+	+	+	+	+
*Hyphopichia fennica*	9849	+	+	+	+ST	+ST	+
*Hyphopichia gotoi*	10145	+	+	+	+ST	+ST	+ST
*Hyphopichia homilentoma*	1507	+ST	+	+	+ST	+ST	-
*Hyphopichia khmerensis*	13262	+ST	+ST	+ST	+	+	+
*Hyphopichia pseudoburtonii*	16346	+	+	+	+ST	+ST	+
*Hyphopichia rhagii*	9839	+ST	+ST	+ST	+ST	+ST	+ST
*Metschnikowia agaves*	31832	+	+	+	+/s	+/s	-
*Metschnikowia kofuensis*	12563	+	+	+/s	+	+	-
*Metschnikowia lunata*	1798	+	+	+	+ST	+ST	-
*Metschnikowia reukaufii*	7534	+	+	+	+	+	-
*Metschnikowia torresii*	1845	+ST	+ST	-	+ST	+ST	-
*Metschnikowia viticola*	12561	+	+	+	+	+	-
**Phaffomycetaceae**							
*Komagataella pastoris*	3650	+ST	+ST	-	+ST	+ST	-
*Phaffomyces opuntiae*	1836	+	+	-	-	- ^1^	- ^1^
*Phaffomyces thermotolerans*	1837	+	+	-	-	- ^1^	- ^1^
**Pichiaceae**							
*Candida ethanolica*	9588	+	+	-	s/-	s/-	-
*Candida inconspicua*	9555	+	+	-	+	+	-
*Candida pseudolambica*	9830	+ST	+ST	-	+ST	+ST	-
*Candida rugopelliculosa*	1593	+	+	-	+ST	+ST	-
*Candida silvatica*	9828	+	+	-	-	-	-
*Dekkera anomala*	31686	+ST	+ST	+	+ST	+ST	+
*Dekkera bruxellensis*	11407	+	+	+	+ST	+ST	+ST
*Kregervanrija fluxuum*	3646	+	+	-	+/s	+/s	-
*Pichia cactophila*	1830	+	+	-	+/s	+/w	-
*Pichia exigua*	1829	+	+	-	w	w	-
*Pichia heedii*	1833	+	+	-	+/w	+/w	-
*Pichia kluyveri* var. *kluyveri*	11403	+	+	-	+ST	+ST	-
*Pichia membranifaciens*	1442	+	+	-	w	w	-
*Pichia myanmarensis*	12922	+ST	+ST	+ST	+ST	+ST	+
*Pichia nakasei*	1699	+ST	+ST	-	+ST	+ST	-
*Pichia occidentalis*	1711	+ST	+	-	+ST	+ST	-
*Pichia rarassimilans*	14993	+	+	-	s	s	-
*Pichia terricola*	1709	+ST	+	-	+	+	-
*Saturnispora ahearnii*	10726	+	+ST	-	+ST	+ST	-
*Saturnispora besseyi*	1706	+ST	+	-	+ST	+ST	-
*Saturnispora dispora*	1795	+	+	-	+ST	+ST	-
*Saturnispora diversa*	1848	+	+	-	+ST	+ST	-
*Saturnispora saitoi*	1793	+ST	+ST	-	+ST	+ST	-
*Saturnispora silvae*	6352	+ST	+	-	+/s	+/s	-
*Saturnispora zaruensis*	1515	+ST	+	-	+ST	+ST	-
**Saccharomycetaceae**							
*Candida castellii*	9550	+	+	-	+ST	+ST	-
*Candida glabrata*	3761	+	+	-	+ST	+ST	-
*Issatchenkia orientalis*	1710	+ST	+ST	-	+ST	+ST	-
*Kazachstania aerobia*	31691	+	+	-	+ST	+ST	-
*Kazachstania bulderi*	31689	+	+	+	+ST	+ST	+
*Kazachstania exigua*	1790	+	+	+	+ST	+ST	+
*Kazachstania humilis*	9852	+	+	-	+ST	+ST	-
*Kazachstania servazzii*	5179	+	+	-	+ST	+ST	-
*Kazachstania telluris*	5298	+	+	-	+ST	+/w	-
*Kazachstania transvaalensis*	5178	+	+	-	+ST	+ST	-
*Kazachstania unispora*	5180	+	+	-	+ST	+ST	-
*Kluyveromyces marxianus*	9556	+	+	+	+ST	+ST	+ST
*Kluyveromyces nonfermentans*	10232	+	+	-	-	-	-
*Lachancea kluyveri*	7257	+	+	+	+ST	+ST	+ST
*Lachancea thermotolerans*	19085	+	+	+	+ST	+ST	+
*Lachancea waltii*	10745	+	+	+	+ST	+ST	+ST
*Saccharomyces bayanus*	7258	+	+	+	+ST	+ST	+ST
*Saccharomyces cerevisiae*	7255	+	+	+	+ST	+ST	+ST
*Saccharomyces pastorianus*	7256	+	+	+	+ST	+ST	+ST
*Tetrapisispora arboricola*	10813	+	+	-	+ST	+ST	-
*Tetrapisispora iriomotensis*	10810	+	+	-	+ST	+ST	-
*Tetrapisispora namnaoensis*	12664	+	+	-	+ST	+ST	-
*Tetrapisispora nanseiensis*	10811	+	+	-	+ST	+ST	-
*Torulaspora delbrueckii*	31684	+	+	-	+ST	+ST	-
*Torulaspora pretoriensis*	3662	+	+	+	+ST	+ST	+
*Zygosaccharomyces rouxii*	7619	+	+	w/-	+ST	+ST	s
*Zygosaccharomyces rouxii*	22060	+	+	-	+ST	+ST	-
*Zygosaccharomyces siamensis*	16825	+	+	-	+ST	+ST	s/w
*Zygotorulaspora mrakii*	1800	+	+	+	+ST	+ST	+
**Saccharomycodaceae**							
*Hanseniaspora opuntiae*	31690	+	+	-	+ST	+ST	-
**Saccharomycopsidaceae**							
*Candida fragicola*	1589	+	+	-	+ST	+ST	-
*Saccharomycopsis capsularis*	7619	+	+	w/-	+ST	+ST	s
*Saccharomycopsis crataegensis*	1700	+	+	-	+	+	-
*Saccharomycopsis fibuligera*	7609	+	+	+	+ST	+ST	+
*Saccharomycopsis javanensis*	3707	+	+	-	-	- ^1^	- ^1^
*Saccharomycopsis malanga*	7620	+	+	-	+/s	+	-
*Saccharomycopsis selenospora*	7616	+	+	-	-	-	-
*Saccharomycopsis synnaedendra*	7607	+	+	-	-	- ^1^	- ^1^
*Saccharomycopsis vini*	7623	+	+	+	+	+	+
**Trichomonascaceae**							
*Blastobotrys adeninivorans*	8914	+ST	+ST	+ST	+ST	+	+/s
*Blastobotrys arbuscula*	2926	+	+	-	+ST	+	-
*Blastobotrys aristata*	2929	+	+	s	+ST	+ST	-
*Blastobotrys capitulata*	2934	+	+	-	+ST	+ST	-
*Blastobotrys chiropterorum*	9597	+	+	+	-	-	-
*Blastobotrys elegans*	2931	+	+	-	+	+/w	-
*Blastobotrys gigas*	2927	+	+	-	+ST	+	-
*Blastobotrys nivea*	2933	+	+	s	+	+	-
*Blastobotrys parvus*	9487	+	+	s	-	-	-
*Blastobotrys proliferans*	2928	+	+	+	+	+	-
*Blastobotrys terrestris*	8913	+	+	+	-	-	-
*Candida santjacobensis*	8924	+	+	+	+	+	-
*Groenewaldozyma auringiensis*	9593	+	+	-	+ST	+	-
*Groenewaldozyma salmanticensis*	8896	+	+	+	+ST	+ST	+ST
*Middelhovenomyces petrohuensis*	8922	+	+	+	-	-	-
*Middelhovenomyces tepae*	10265	+	+	s	w	-	-
*Sugiyamaella castrensis*	9585	+	+	+	w/-	-	-
*Sugiyamaella paludigena*	9614	+	+	+	-	-	-
*Sugiyamaella valdiviana*	9565	+	+	+	+/w	w/-	-
*Trichomonascus ciferrii*	7621	+	+	+	-	-	-
*Wickerhamiella azyma*	1691	+	+	+	-	-	-
*Wickerhamiella domercqiae*	9478	+	+	+/w	-	- ^1^	- ^1^
*Wickerhamiella galacta*	8257	+	+	-	-	-	-
*Wickerhamiella hasegawae*	12559	+	+	-	+	+/w	-
*Wickerhamiella kazuoi*	12558	+	+	-	-	-	-
*Wickerhamiella pararugosa*	1512	+	+	-	-	- ^1^	- ^1^
*Wickerhamiella sorbophila*	1514	+	+	-	-	-	-
*Wickerhamiella spandovensis*	9562	+	+	+	+ST	w	w
*Wickerhamiella vanderwaltii*	9615	+	+	-	-	-	-
*Wickerhamiella versatilis*	8065	+	+	+	+ST	+ST	+ST
*Zygoascus biomembranicola*	31007	+	+	-	+ST	+	-
**Wickerhamomycetaceae**							
*Barnettozyma salicaria*	3653	+	+	-	-	-	- ^1^
*Barnettozyma wickerhamii*	21961	+ST	+ST	+ST	+	+	-
*Candida berthetii*	9594	+	+	-	+	+	-
*Candida danieliae*	17247	+ST	+ST	+	+ST	+	-
*Candida dendrica*	9605	+	+	-	+	+/s	-
*Candida easanensis*	12476	+	+	+/s	+	+	-
*Candida eppingiae*	17241	+ST	+ST	+ST	+ST	+ST	-
*Candida freyschussii*	9850	+	+	+/s	+	+	-
*Candida maritima*	9612	+	+	+	+	+	+
*Candida montana*	2323	+	+	-	-	- ^1^	- ^1^
*Candida nakhonratchasimensis*	12474	+	+	+	+ST	+ST	+ST
*Candida norvegica*	8897	+	+	-	-	-	-
*Candida pattaniensis*	12475	+ST	+ST	+ST	+ST	+ST	+
*Candida pseudoflosculorum*	17242	+ST	+ST	+ST	+ST	+ST	+ST
*Candida quercuum*	1587	+ST	+	+	+/w	+	-
*Candida robnettiae*	17243	+ST	+ST	+ST	+ST	+ST	-
*Candida silvicultrix*	9831	+	+	+	+ST	+ST	+ST
*Candida solani*	2339	+	+	+	+ST	+ST	-
*Candida vartiovaarae*	3759	+	+	+	+ST	+ST	+
*Cyberlindnera americana*	3592	+	+	+	-	+/s	-
*Cyberlindnera americana*	3593	+	+	+	w	s	-
*Cyberlindnera amylophila*	1702	+	+	+	+ST	+ST	-
*Cyberlindnera bimundalis*	3591	+	+	+	+/s	+/s	-
*Cyberlindnera fabianii*	3601	+ST	+	+	+ST	+ST	+ST
*Cyberlindnera jadinii*	3617	+	+	+	+ST	+ST	+ST
*Cyberlindnera japonica*	11402	+	+	+	s	s/w	-
*Cyberlindnera mississippiensis*	1703	+ST	+	+	+ST	+ST	-
*Cyberlindnera mrakii*	3614	+	+	-	+ST	+ST	-
*Cyberlindnera petersonii*	3619	+	+	+	+	+	+
*Cyberlindnera rhizosphaerae*	16499	+	+	s	+	+	+
*Cyberlindnera rhodanensis*	3649	+ST	+ST	+ST	+ST	+ST	-
*Cyberlindnera samutprakarnensis*	17816	+	+	+	+ST	+ST	+ST
*Cyberlindnera subsufficiens*	3625	+	+	+	+	+	+
*Starmera amethionina*	1831	+	+	-	s	s	-
*Starmera pachycereana*	1832	+	+	-	-	-	-
*Starmera quercuum*	3659	+	+	-	+	+	-
*Starmera stellimalicola*	3546	+	+	-	+ST	+ST	-
*Wickerhamomyces anomalus*	3585	+ST	+ST	+ST	+ST	+ST	+ST
*Wickerhamomyces bisporus*	3590	+	+	+	w	w	-
*Wickerhamomyces bovis*	3640	+ST	+ST	+ST	+ST	+ST	-
*Wickerhamomyces canadensis*	3597	+ST	+ST	+ST	-	- ^1^	- ^1^
*Wickerhamomyces chaumierensis*	17246	+ST	+ST	+ST	+ST	+ST	-
*Wickerhamomyces ciferrii*	3599	+ST	+ST	+ST	+	+ST	+
*Wickerhamomyces mucosus*	6814	+	+	+	+ST	+ST	-
*Wickerhamomyces patagonicus*	16381	+	+	-	-	-	-
*Wickerhamomyces pijperi*	11406	+	+	-	+ST	+ST	-
*Wickerhamomyces silvicola*	3627	+ST	+ST	+	+ST	+ST	-
*Wickerhamomyces subpelliculosus*	3631	+ST	+ST	+	+ST	+ST	+ST
*Wickerhamomyces sydowiorum*	9455	+ST	+ST	+ST	+	+ST	+
**Saccharomycetales *incertae sedis***							
*Ambrosiozyma cicatricosa*	7598	+ST	+	+	+/s	+/s	+/s
*Ambrosiozyma kamigamensis*	14990	+	+	+/s	+	+	-
*Ambrosiozyma kashinagicola*	15019	+	+	-	+	+	-
*Ambrosiozyma llanquihuensis*	8918	+	+	-	+	+	-
*Ambrosiozyma monospora*	7599	+	+	+	+ST	+ST	+/w
*Ambrosiozyma neoplatypodis*	14992	+	+	-	+	+	-
*Ambrosiozyma oregonensis*	1797	+ST	+	+	+	+	-
*Ambrosiozyma philentoma*	7600	+	+	+	+/d	+	d/w
*Ambrosiozyma platypodis*	1843	+	+	+	w	+	-
*Ambrosiozyma platypodis*	1796	+	+	+	+/s	+	-
*Ambrosiozyma pseudovanderkliftii*	15025	+	+	s	+	+	-
*Ambrosiozyma vanderkliftii*	15029	+ST	+ST	+	+ST	+ST	w
*Babjeviella inositovora*	10736	+	+	+	-	-	-
*Candida arabinofermentans*	10727	+	+	-	+	+	-
*Candida blankii*	8259	+	+	+	+/s	w	w
*Candida boidinii*	9604	+	+	-	+ST	+ST	-
*Candida chilensis*	1693	+	+	+	+	+	-
*Candida cylindracea*	9586	+	+	-	+	+	-
*Candida digboiensis*	12330	+	+	+	-	-	-
*Candida entomophila*	9607	+	+	+	+ST	+ST	+ST
*Candida incommunis*	8258	+	+	+	+	+	-
*Candida insectalens*	9610	+	+	-	-	-	-
*Candida krabiensis*	12266	+	+	-	-	-	-
*Candida maris*	9853	+	+	-	-	-	-
*Candida methanosorbosa*	9620	+	+	-	+	+	-
*Candida nanaspora*	9590	+	+	-	+ST	+ST	-
*Candida nemodendra*	9855	+	+	-	s/-	w/-	-
*Candida nitratophila*	9856	+	+	-	+	+	-
*Candida ovalis*	9444	+	+	-	+ST	+ST	-
*Candida pini*	9826	+	+/s	-	+/s	+/s	-
*Candida sake*	2951	+ST	+	+	+ST	+ST	-
*Candida savonica*	9561	+	+	-	+	+	-
*Candida sequanensis*	9841	+	+	-	+ST	+ST	-
*Candida silvanorum*	1804	+	+	+	+ST	+ST	-
*Candida sithepensis*	12265	+	+	-	+ST	+ST	-
*Candida sonorensis*	1827	+	+	-	+ST	+ST	-
*Candida sophiae-reginae*	8925	+ST	+ST	+	+ST	+ST	-
*Candida sorboxylosa*	1536	+ST	+ST	-	+	+	-
*Candida succiphila*	9445	+	+	-	+ST	+ST	-
*Citeromyces matritensis*	2333	+	+	+	+ST	+ST	+ST
*Citeromyces siamensis*	11522	+	+	+	+ST	+ST	+ST
*Diutina catenulata*	1604	+ST	+	-	+	+	-
*Diutina rugosa*	1619	+ST	+	-	-	- ^1^	- ^1^
*Kuraishia capsulata*	1991	+	+	-	+	+	-
*Nadsonia commutata*	10138	+	+	-	-	- ^1^	- ^1^
*Nadsonia fulvescens* var. *fulvescens*	9992	+	+	-	+ST	+ST	-
*Nadsonia starkeyi-henricii*	11408	+	+	-	-	-	-
*Nakazawaea anatomiae*	9547	+	+	-	+	+	-
*Nakazawaea holstii*	3608	+	+	+/s	+ST	+ST	-
*Nakazawaea ishiwadae*	9451	+	+	+	+ST	+ST	-
*Nakazawaea peltata*	9829	+	+	+	+ST	+ST	-
*Nakazawaea populi*	9833	+	+	s	+ST	+ST	-
*Nakazawaea wickerhamii*	9568	+	+	-	+ST	+ST	-
*Ogataea angusta*	3635	+	+	+	+ST	+ST	-
*Ogataea glucozyma*	3607	+	+	-	+ST	+ST	-
*Ogataea henricii*	3611	+	+	-	-	- ^1^	- ^1^
*Ogataea kodamae*	11404	+	+	-	+/s	+/s	-
*Ogataea methanolica*	10240	+	+	-	+ST	+ST	-
*Ogataea methylivora*	22142	+	+	+	+	+	-
*Ogataea minuta*	3622	+	+	-	+	+ST	-
*Ogataea naganishii*	22078	+	+	+	+	+	-
*Ogataea nonfermentans*	3615	+	+	-	-	-	- ^1^
*Ogataea philodendri*	22070	+	+	-	-	-	-
*Ogataea pignaliae*	9836	+	+	-	+ST	+ST	-
*Ogataea pini*	3655	+	+	-	s/-	s/-	-
*Ogataea salicorniae*	10744	+	+	-	+ST	+ST	-
*Ogataea siamensis*	12264	+	+	+	+/s	+/s	-
*Ogataea thermomethanolica*	12984	+	+	+	+	+	-
*Ogataea trehalophila*	3651	+ST	+ST	-	+ST	+ST	-
*Pachysolen tannophilus*	31685	+	+	-	+ST	+ST	-
*Peterozyma toletana*	3658	+ST	+	+	+	+	-
*Saprochaete japonica*	2451	+ST	+ST	-	+ST	+ST	-
*Sporopachydermia cereana*	9480	+	+	-	-	-	-
*Sporopachydermia lactativora*	9485	+	+	-	-	- ^1^	- ^1^
*Sporopachydermia quercuum*	9486	+	+	-	+	-	-
*Starmerella apicola*	9592	+	+	+	+	+	+
*Starmerella apis*	8256	+ST	+ST	+	-	w	-
*Starmerella bombi*	9595	+	+	+	+ST	+ST	+
*Starmerella bombicola*	9596	+	+	+	+ST	+ST	+ST
*Starmerella etchellsii*	8066	+	+	-	+	s/w	-
*Starmerella floricola*	9439	+	+	+	+ST	+ST	+ST
*Starmerella geochares*	9851	+	+	+	+	+	+/s
*Starmerella gropengiesseri*	8255	+	+	+	+	+	w
*Starmerella lactis-condensi*	9472	+	+	+	+ST	+ST	+ST
*Starmerella magnoliae*	1446	+	+	+	+	+	+
*Starmerella stellata*	9476	+	+	+	+ST	+ST	+ST
*Starmerella vaccinii*	9446	+	+	+	+	+	+
*Suhomyces tanzawaensis*	1648	+	+	+	s/w	+/w	-
*Teunomyces kruisii*	1779	+	+	+	+ST	+ST	-
*Trigonopsis cantarellii*	8260	+	+	-	+ST	+ST	-
*Trigonopsis variabilis*	1823	+	+	-	-	- ^1^	- ^1^
*Trigonopsis vinaria*	1813	+	+	-	-	- ^1^	- ^1^
*Yarrowia deformans*	1694	+	+	-	-	- ^1^	- ^1^
*Yarrowia keelungensis*	14894	+ST	+ST	-	-	-	-
*Yarrowia lipolytica*	2320	+ST	+ST	-	-	- ^1^	- ^1^
*Yarrowia yakushimensis*	12782	+ST	+ST	-	-	-	-
**Taphrinomycotina**							
*Saitoella complicata*	7358	+	+	+	-	-	-
*Schizosaccharomyces japonicus*	8264	+	+	+	+ST	+ST	+ST
*Schizosaccharomyces octosporus*	8261	+	+	w	+	+	w
*Schizosaccharomyces pombe*	8274	+	+	+	+ST	+ST	+ST
**Pezizomycotina**							
*Trichosporiella flavificans*	1506	+	+	-	+ST	+ST	-

^1^ Fermentation test examined once; +ST, strongly positive; +, positive; d, delayed positive; s, slowly positive; w, weakly positive; -, negative; a diagonal line “/” indicates “or”.

**Table 2:** Percentage of yeast species in the Ascomycota capable of assimilating/fermenting common sugars.

Sugars	This Study		The Yeasts *	
	Assimilation	Fermentation	Assimilation	Fermentation
Glucose	100% (388/388)	77.8% (302/388)	100% (827/827)	72.8% (602/827)
Fructose	100% (388/388)	77.8% (302/388)	nd	nd
Sucrose	59.0% (229/388)	25.5% (99/388)	60.7% (502/827)	24.2% (200/827)
Galactose	nt	nt	65.4% (541/827)	30.6% (253/827)
Trehalose	nt	nt	70.0% (579/827)	30.1% (249/827)
Maltose	nt	nt	56.8% (470/827)	18.3% (151/827)
Raffinose	nt	nt	28.5% (236/827)	13.8% (114/827)

* Data collected from “*The Yeasts, a Taxonomic Study, 5th edition*” [3]; “v (variable)” counted as positive; the percentages were calculated on the number of species basis. nt, not tested; nd, no data.

## Data Availability

Data supporting results can be found in Supplementary Table S1.

## Supplementary Materials

The following are available online at https://www.mdpi.com/article/10.3390/microorganisms9040758/s1, Figure S1: Gas filling in a Durham tube in the fermentation liquid medium, Table S1: (a) Growth in the assimilation media containing each sugar, (b) Filling of gas in Durham tube in the fermentation media containing each sugar.
