# Supplementary figures and images for: D-Fructose Assimilation and Fermentation by Yeasts Belonging to Saccharomycetes: Rediscovery of Universal Phenotypes and Elucidation of Fructophilic Behaviors in Ambrosiozyma platypodis and Cyberlindnera americana
Source: Microorganisms. 2021 Apr 5;9(4):758. doi: 10.3390/microorganisms9040758 (PMC8065679; doi:10.3390/microorganisms9040758)

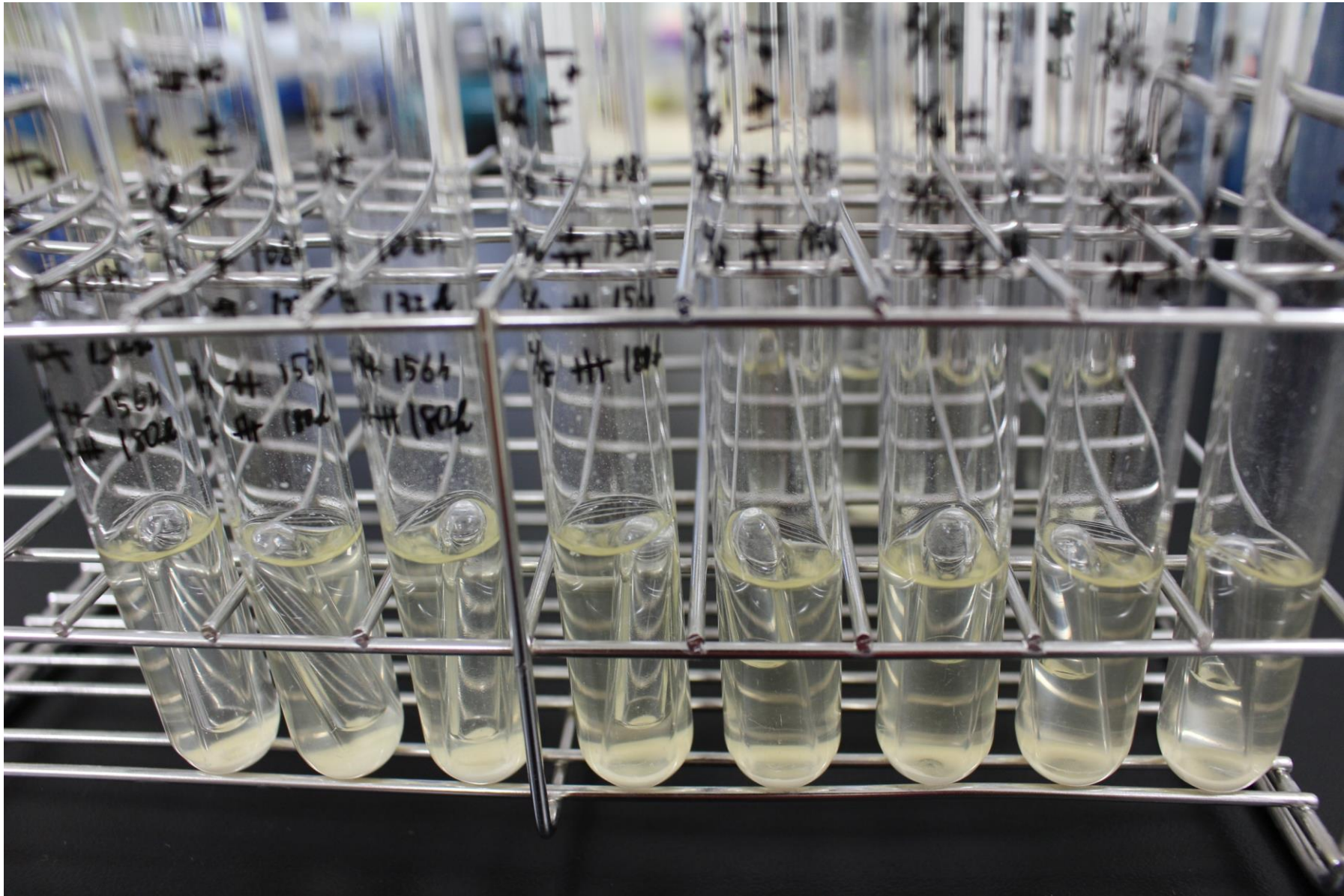

**Figure S1.** Gas filling in a Durham tube in the fermentation liquid medium.

Supplement: Supplementary file 1 [file microorganisms-09-00758-s001.zip › microorganisms-1050919 -SUP/microorganisms-1050919-SupplementaryFigure1.pdf]
